# Environmental enrichment reverses prenatal ethanol exposure-induced attention-deficits in rats

**DOI:** 10.3389/fpsyt.2025.1549318

**Published:** 2025-03-26

**Authors:** Ruixiang Wang, Connor D. Martin, Anna L. Lei, Kathryn A. Hausknecht, Jerry B. Richards, Samir Haj-Dahmane, Roh-Yu Shen

**Affiliations:** Department of Pharmacology and Toxicology, University at Buffalo, The State University of New York, Buffalo, NY, United States

**Keywords:** attention deficit-hyperactivity disorder, anxiety, fetal alcohol spectrum disorders, impulsivity, sustained attention

## Abstract

**Introduction:**

There is a high prevalence of fetal alcohol spectrum disorders (FASD) in the US and the world, which is caused by prenatal ethanol exposure (PE). Most individuals with FASD show attention deficit hyperactivity disorder (ADHD) -like symptoms. Using a rat model of FASD, we have successfully demonstrated that moderate and heavy PE leads to persistent attention deficits, including augmented impulsivity and impaired sustained attention. Anxiety is another primary symptom of FASD. Anxiety and ADHD are closely associated in clinical studies. However, the causal relationship between anxiety and ADHD is not clear. In the present study, we used the strategy of environmental enrichment to reduce anxiety after PE in rats and investigated if attention deficits could be ameliorated.

**Methods:**

A 2nd-trimester binge-drinking pattern of heavy PE was used. Environmental enrichment consisted of neonatal handling and postweaning complex housing. Action impulsivity and sustained attention were tested in adult males and females using the 2-choice reaction time task.

**Results:**

The results show environmental enrichment effectively ameliorated action impulsivity and improved sustained attention in male and female PE rats. Action impulsivity was also improved in control rats with environmental enrichment. In addition, environmental enrichment improved the efficiency of obtaining rewards in male and female control but not PE rats. Environmental enrichment altered the pattern of reaction time components, favoring slower movement initiation but faster movement.

**Discussion:**

These observations support that environmental enrichment could be an effective strategy in ameliorating ADHD-like symptoms in FASD. The reduced anxiety could contribute to such an effect.

## Introduction

As one of the most common developmental disorders, attention-deficit/hyperactivity disorder (ADHD) is associated with a variety of genetic and environmental risk factors. Recent studies elucidate that genetic risk factors, prenatal stress factors and/or postnatal adverse environment all contribute to ADHD (1, 2). Results from preclinical and clinical studies have shown prenatal ethanol exposure (PE) during pregnancy could lead to ADHD-like symptoms (3–8). Prenatal ethanol exposure leads to fetal alcohol spectrum disorders (FASD), which has a high prevalence (2-5%) in the US (9, 10). The incidence of ADHD in individuals diagnosed with fetal alcohol spectrum disorders (FASD) can be as high as 49 – 94% (4, 6, 8). The estimated prevalence of ADHD in the general population is around 5% (11, 12). Therefore, prenatal ethanol exposure could represent a key environmental risk factor for ADHD.

When compared with ADHD cases without FASD, attention deficits in individuals with FASD could differ in severity and/or symptoms (4, 13), have an earlier onset, and have differential responses to stimulant treatment (8, 14). On the other hand, our previous preclinical study demonstrates that chronic psychostimulant treatment used in treating ADHD is effective in normalizing altered dopamine neuron activities, which play a critical role in mediating impulsivity and attention behaviors (15), supporting the efficacy of psychostimulant treatment in attention deficits in FASD.

At the present time, there are few treatment options available using medication for FASD, (14, 16). A variety of cognitive/behavioral deficits could be ameliorated when children with FASD are raised in favorable environments (17), which is consistent with animal studies showing the efficacy of environmental enrichment in alleviating PE-induced behavioral deficits (18–21)and PE-induced neuroinflammation (22). To maximize the effectiveness of environmental enrichment intervention in rats, we have combined neonatal handling and post-weaning complex housing to provide persistent enrichment throughout development (23). This approach has been shown to ameliorate multiple behavioral deficits after PE, including the increased drug addiction risk (20), impaired habituation in sensory processing (21), and augmented anxiety (24). Among all these beneficial effects of environmental enrichment, reduced anxiety could be critical for attention deficits. In clinical studies, attention deficits and anxiety are closely associated (25), but the causal relationship between these two behavioral phenotypes is not clear. Our previous preclinical study demonstrates that increasing trait anxiety exacerbates attention deficits (26), supporting that increased anxiety contributes to attention deficits. Because environmental enrichment can reduce anxiety (20, 27), we anticipate that environmental enrichment could ameliorate ADHD-like symptoms by reducing anxiety. Such a result would further verify that anxiety modulates the severity of attention deficits.

There are two major symptoms of ADHD: inattention and hyperactivity/impulsivity. Subtypes of ADHD could be predominantly inattentive, predominantly hyperactive, or showing combined presentation (DSM-5, 28). The two symptoms could be mimicked in rodent models, typically by tasks including multiple choices and requiring short reaction time (29). In our laboratory, we utilize a 2-choice reaction time (2-CRT) task with variable, challenging hold time requirements, which meets the above criteria. Using this 2-CRT task, we have demonstrated ADHD-like symptoms in rats with heavy and moderate PE (5, 7) as well as rats with increased trait anxiety (26). The training period for the 2-CRT task is relatively brief, which does not confound attention deficit symptoms. As such, we employed the 2-CRT task in the present study to investigate if environmental enrichment affects attention deficits in rats with heavy PE.

## Materials and methods

### Animal breeding and prenatal ethanol exposure

The breeding procedure has been described in detail previously (30). We bred rats in-house to avoid transportation stress during pregnancy. Male Sprague-Dawley breeders and virgin females (Envigo, Indianapolis, IN, USA) were housed in pairs in wire-bottom breeding cages in a 12 h/12 h light/dark cycle room. Gestational day (GD) 0 was designated when copulatory vaginal plugs were found. Pregnant dams were randomly assigned to the control or PE group and singly housed in standard plastic cages.

A second-trimester binge drinking pattern model of PE is used, which is comparable to heavy alcohol exposure in humans (31, 32). From GD 8 – 20, pregnant dams were treated via intragastric gavage twice (5 – 6 h apart) every weekday during the light phase with 3 g/kg ethanol (15% w/v) or isocaloric vehicle (22.5% w/v sucrose). The total dose of ethanol is 6 g/kg/day. A single dose of 4 g/kg ethanol was given on each weekend day. The blood alcohol level 1 hour after the 2nd gavage was 116.8 ± 10.5  mg/dl (30). We chose the gavage procedure to control ethanol dosing precisely. We have shown that stress caused by our gavage procedure is minimal (33). Controls were pair-fed with PE rats to equalize daily nutrient intake during ethanol administration. To prevent possible thiamine deficiency during ethanol exposure or by the pair-feeding procedure, dams received vitamin B injections (8 mg/kg; i.m.; twice a week) during ethanol administration (34, 35).

On postnatal day 1, each litter was randomly culled to 10 pups with 5 males and 5 females. Cross-fostering was performed on PD 1 to minimize the possible alcohol withdrawal effects on maternal behavior, which might impact the rearing of the pups. Ethanol-exposed pups were transferred to foster dams who received no treatment except daily weighing and gave birth 2 days earlier. Control litters were cross fostered by switching the control dams. This way, all litters were cross-fostered. In humans, fostering could have long-lasting effects on mental health in some individuals (36). Results from preclinical studies suggest that cross-fostering could reduce anxiety and PE effects (37, 38). In our lab, we do not observe any difference in body weight at weaning between fostered and non-fostered pups in either control or PE groups (unpublished data). On PD 21, litters were weaned, and same-sex rats were housed in pairs in standard cages. One hundred fifty-five rats from 42 litters were used in the 2-CRT test (24 control males in standard condition/8 litters, 24 PE males in standard condition/9 litters, 16 control females in standard condition/6 litters, & 15 PE females in standard condition/6 litters, 20 control males in the enriched condition/7 litters, 20 PE males in enriched condition/8 litters, 18 control females in enriched condition/5 litters, & 18 PE females in enriched condition/5 litters). In 6 control litters and 6 PE litters, both sexes were used. All the animal-related procedures followed the guidelines of the National Institutes of Health regarding laboratory animal care and use and were approved by the Institutional Animal Care and Use Committee of the University at Buffalo.

### Rearing conditions

Rats were reared in the standard housing condition or enriched condition. Before weaning, pups reared in the standard condition were not disturbed except for weekly cage changes. All pups reared in the enriched condition underwent neonatal handling consisting of a brief (15 min/day) maternal separation and handling of each pup from postnatal day 2 - 20. The goal was to provide enrichment by enhancing maternal behavior when pups were reunited with the dam (20, 21, 39–41). After weaning (postnatal day 21), rats in the standard housing condition were housed in pairs in standard plastic cages. Rats in the enriched condition were group housed (10 – 20/cage) with the same sex and prenatal treatment in large 4-level wire cages (L × W × H: 92 × 64 × 160 cm; Model: CG-71111; Petco, San Diego, CA, USA). Each cage contained 30 pet toys, ropes, hideouts, etc./cage (Petco), which were moved or changed every weekday to create novelty (see more details in (20). The rearing conditions were maintained until the completion of the study.

### Apparatus

Sixteen locally made operant chambers were used, which were described in detail previously (3, 42). In the right wall panel, there were two water dispensers, each inside a snout poke hole on either side of a centrally located snout-poke hole. There was a stimulus light above each of the water dispensers. Snout pokes into the snout poke holes were monitored with infrared sensors. A drop of water (0.03 ml/drop), as a reinforcer was delivered into dispensers by a syringe pump (PHM-100; MED Associates, Fairfax, VT, USA). All chambers were controlled by MED Associates interface and software.

### 2-CRT task

A modified 2-CRT task was used. Six-week-old rats were water-restricted (water available for 0.5 h/day), so water served as a reinforcer. The rats underwent *18* daily 30 min training sessions and 3 additional 30 min testing sessions during the dark phase. A trial was initiated by the rat inserting its snout into the center hole. The rat was required to hold the snout in the center hole for a predetermined period (hold time) until one of the stimulus lights turned on. The hold time was cumulative for the duration each time the rat put the snout in; no matter how many times the rat pulled out of the center hole. When the hold time was up, either the left or right stimulus light was turned on. The rat would obtain the water reward only when a correct trial was made - enter into the poke hole/water dispenser under the lit stimulus light - in a timely manner. The time elapsed between snout withdrawal from the center hole and entering the water dispenser was defined as total RT, which consisted of initiation time - the time between the stimulus light onset and snout withdrawal from the center hole - and movement time - the time between snout withdrawal from the center hole and entering the water dispenser. An incorrect trial (entering the water dispenser not associated with the lit stimulus light) would terminate the trial immediately, with no water delivery. An extremely slow response (i.e., when RT > maximal trial duration, which was 2 s in the final testing sessions) was considered an omission, leading to no water reward. Choice trials consisted of correct trials, incorrect trials, and omissions.

Forced trials (training trials) were also programmed to avoid spontaneous alteration (43) and facilitate correct responses. After an incorrect trial, a forced trial took place. The trial with the same stimulus light was repeated until the rat chose the right water dispenser. This last correct trial was reinforced but still counted as a forced trial.

Rats were also trained to respond in a timely way. To that end, variable criterion RTs (maximal RT allowed) for reinforcement were used (3). In each choice trial, if the actual RT > the set criterion RT, no reward was given. If 2 correct responses were made in a row under a specific RT, the criterion RT would decrease for the subsequent trial. If 1 incorrect or slow response (without reinforcement) occurred, the criterion RT would increase for the subsequent trial. The training could accommodate both fast- and slow-responders. The decrement/increment schedule (in seconds) was 27.00, 10.00, 5.00, 2.50, 1.00, 0.89, 0.79, 0.71, 0.63, 0.56, 0.50, 0.45, 0.40, 0.35, 0.32, 0.28, 0.25, 0.22, 0.20, 0.18, 0.16, 0.14, 0.13, 0.12, 0.11, & 0.01. At the beginning of a session, the criterion RT was set at 0.71 s. Under the adjustments of criterion RTs, the goal was for rats to obtain 60% to 75% reinforcements for correct trials.

The rats’ behavior was trained in stages. In sessions 1-2, water was also available in the center hole contingent upon snout pokes. For sessions 1-8, the stimulus light would turn on only one side for all trials. From session 9, the stimulus light would illuminate on the left or the right side randomly. The hold time also increased gradually and transitioned from a fixed length to a variable length with each session. In the final 3 testing sessions, 20 different hold times were used with the mean hold time = 6 s (in seconds: 0.0798, 0.246, 0.4212, 0.6066, 0.8034, 1.0134, 1.2414, 1.4814, 1.7466, 2.031, 2.3466, 2.697, 3.0918, 3.5436, 4.071, 4.7052, 5.514, 6.5712, 8.22, & 12.5868). In addition, the duration of stimulus light in each trial decreased from 3600 s to 1 s. The maximal RT allowed decreased from 3600 s to 2 s.

### Dependent variables

**Figure 1:**
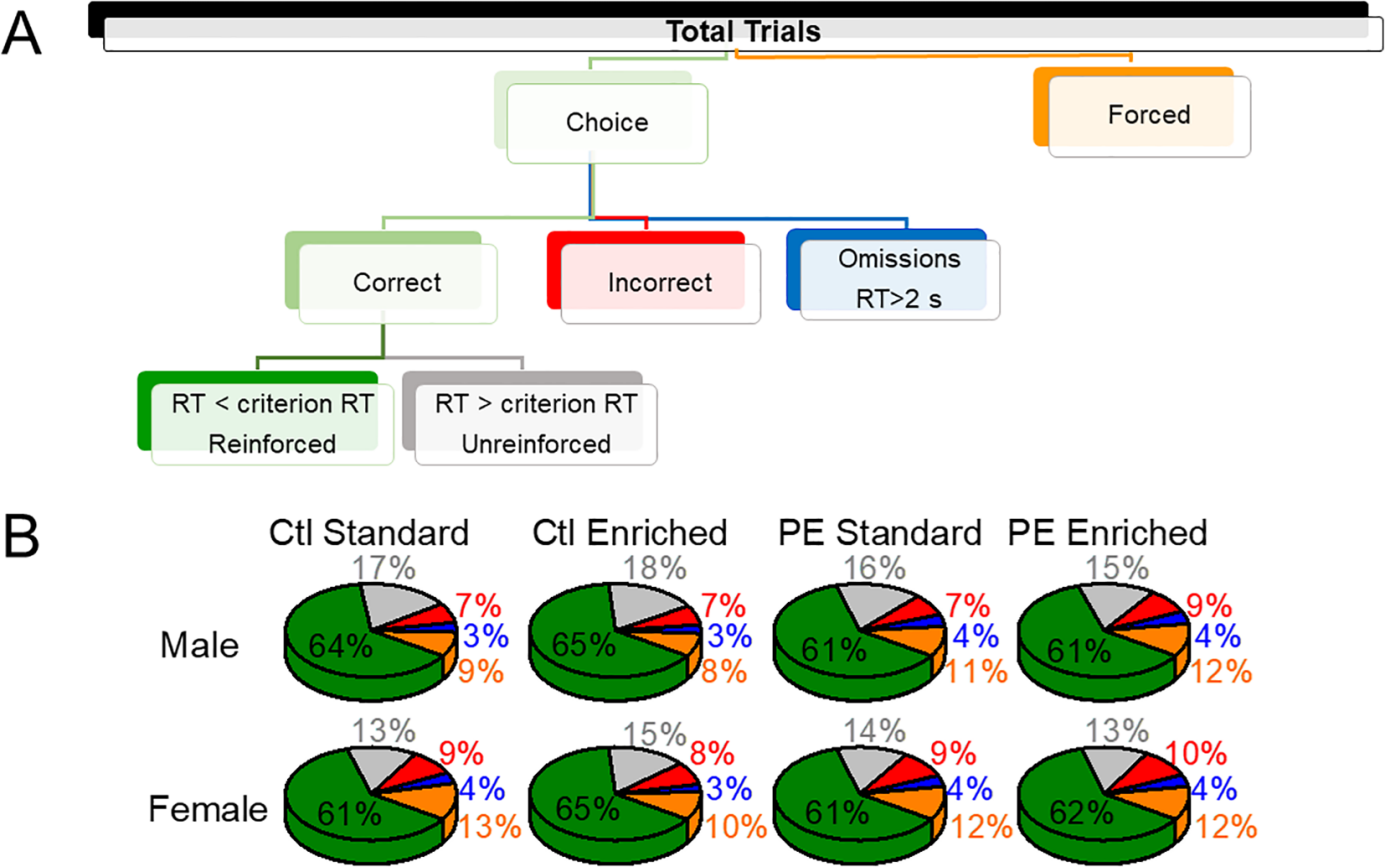
The trial composition of the 2-choice reaction time (2-CRT) task. The upper panel depicts the composition of all trials **(A)**. The lower panel depicts the portions of each trial type **(B)**. Prenatal treatment (control; PE), postnatal rearing conditions (standard; environmental enrichment), or sex (male; female) did not impact the proportions of different trial types.


To evaluate overall performance and possible deficits in operant learning, the number of choice trials, forced trials, and reinforced trials were analyzed (**Figure 1**). Premature responses, including premature initiations and false alarms, were used to assess action impulsivity. Premature initiations occurred when rats repeatedly withdrew the snout from the center hole and reinserted it before the stimulus light turned on. False alarms occurred when rats entered the water dispenser before the stimulus light turned on. Lapses of attention lead to wrong or slow responses, accessed by % incorrect responses over choice trials and % omissions over choice trials. In addition, infrequent long RTs were measured by the skewness of the RT distribution because they lead to the positively skewed distribution of RTs. Infrequent long RTs are also observed in individuals with ADHD (44). In the present study, we used an *adjusted Fisher-Pearson standardized moment* coefficient for skewness, as computed by the following formula (45):

$$skewness=n\frac{\sum^{\;}{\left(X_{i}-Mean_{i}\right)}^{3}}{\left(n-1\right)\left(n-2\right)*\sigma^{3}}$$

where σ was the standard deviation, and *n* was the number of trials (5, 7).

### Data analysis

All the analyses were based on the 3 final testing sessions. Rats were removed from data analysis if the average number of trials/sessions was consistently< 75 for males or< 60 for females. The criterion was lower for females because female rats had lower body weights than males (by 20 – 35%), and thus, they consumed less water.

Two-way or three-way mixed-design or non-mixed design analyses of variances (ANOVA) were used. We applied statistical methods to control for possible litter effects when more than 2 littermates were used in the same sex per group. Litter as a nested factor was used in ANOVA to examine possible litter effects. (21, 41, 46). If a significant litter effect was found, then results from the ANOVA with litter as a nested factor were reported. If the litter effect was not significant, then results from ANOVAs without using litter as a nested factor were reported. Pairwise comparisons were performed using planned comparisons after ANOVA. Statistica 7 (Tibco Software Inc., Palo Alto, CA, USA) software was used for data processing and analysis. The significance level was set at 0.05. Data are presented as Mean ± SEM in the text and figures unless specified otherwise.

## Results

### Birth outcome after PE

Twenty control (11 standard and 9 enriched) and 22 PE (13 standard and 9 enriched) dams were used in this study. Prenatal ethanol exposure did not lead to a reduction in litter size or number of male pups (**Table 1**). However, PE led to fewer female pups/litter (**Table 1**). In addition, the greater body weight of PD 1 was observed in males compared to females regardless of prenatal treatment (t86 = 2.48, p<0.05; (**Table 1**)The results showed that PE did not lead to major teratogenic effects.

**Table 1:** Birth outcome of control and prenatal ethanol-exposed litters.

	Control: 20 litters 11 Std & 9 Enriched (mean ± SEM)	PE: 22 litters 13 Std & 9 Enriched (mean ± SEM)	*P*-Value
Litter Size	13.90 ± 0.49	13.05 ± 0.39	0.18
Number of Male Pups	6.50 ± 0.44	7.05 ± 0.39	0.38
Number of Female Pups	7.40 ± 0.49	6.00 ± 0.37	<0.05
Pup weight on Postnatal Day 1			
Average Weight (g)	6.50 ± 0.14	6.63 ± 0.09	0.45
Average Male Weight (g)	6.68 ± 0.15	6.76 ± 0.09	0.66
Average Female Weight (g)	6.34 ± 0.14	6.44 ± 0.12	0.56

### Effects of PE, sex, and enriched environment on trial performance

**Figure 2:**
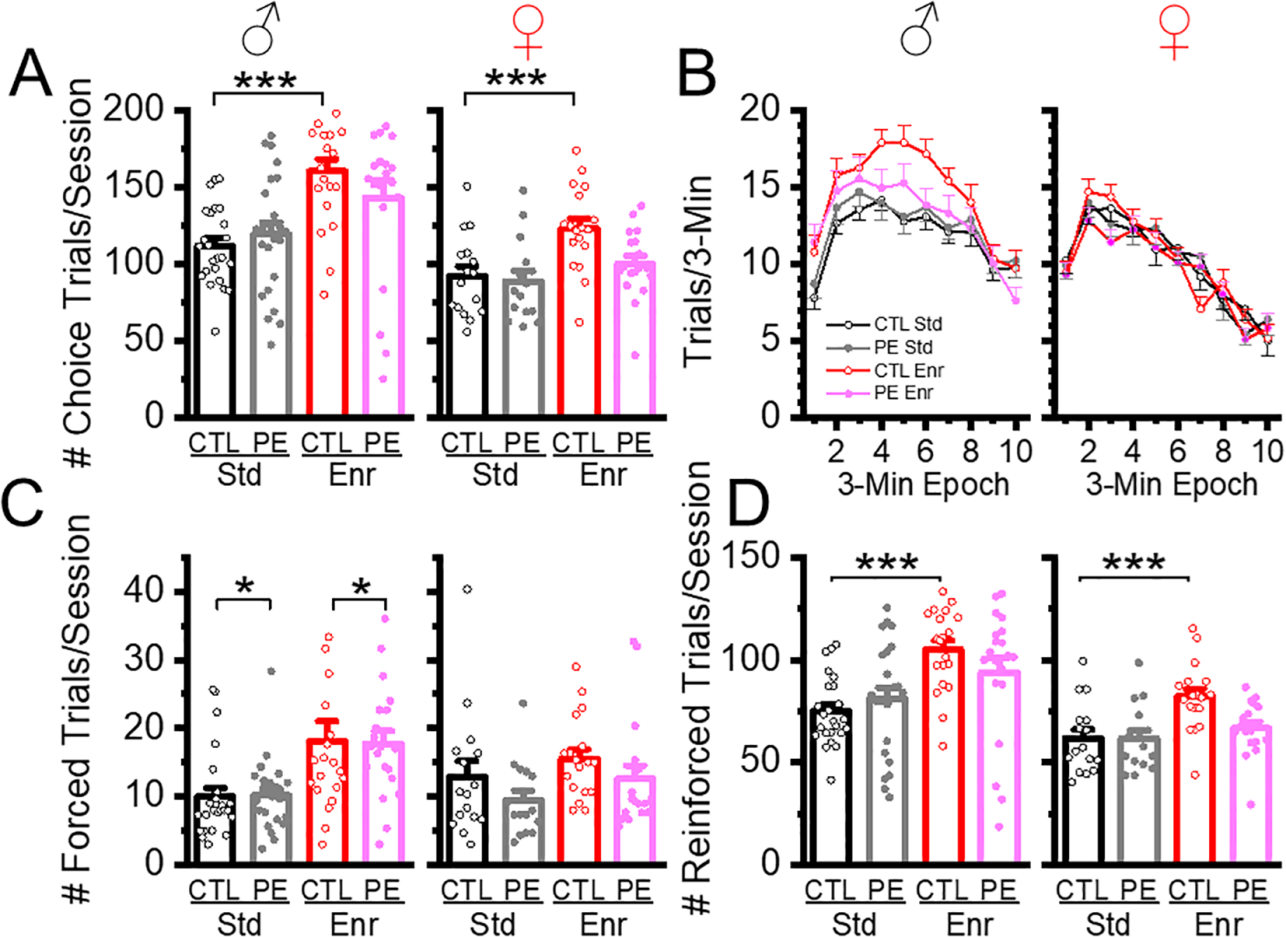
Environmental enrichment increased the number of trials in control and PE rats of both sexes. Rearing in the enriched environment increased the number of choice trials/sessions in control rats of both sexes but not in PE rats **(A)**. Such an effect was more prominent in males, shown as a persistent escalation of trials soon after the session started **(B)**. Environmental enrichment also increased the number of forced trials in male control and PE rats **(C)** and led to more reinforced trials in control, but not PE rats of both sexes **(D)**. *P< 0.05, ***P< 0.001.

The compositions of trials are depicted in **Figure 1**. We examined the effects of PE and rearing conditions on trial composition with a focus on choice trials, forced trials, and reinforced trials. Males performed more trials than females, which is consistent with previous studies (5). In addition, male and female control rats reared in the enriched condition performed more choice trials than rats in the standard condition (**Figure 2A**). Such an effect was not observed in PE rats reared in the enriched condition (three-way way ANOVA with litter as the nested factor, prenatal treatment, postnatal rearing condition, sex; litter effect: F46,101 = 3.99, P<0.01; main effect of sex: F1, 101 = 19.43, P<0.01; interaction effect of prenatal treatment and rearing conditions F1,101 = 17.41, P<0.01; planned comparison: male or female: control stand *vs* control enriched, P<0.001, **Figure 2A**). The effects were caused by control rats in the enriched condition escalated more in their choice trials soon after the session started and sustained such escalation for a long duration. This effect was more prominent in males (**Figure 2**).

We also examined the proportion of forced trials (% forced trials) as an index of possible learning deficits and found male PE rats showed greater % forced trials than controls in both standard and enriched conditions, suggesting rigidity or inability to switch to the correct choice (three-way ANOVA with litter as the nested factor, litter effect, F46,101 = 1.59, P<0.05; interaction effect of prenatal treatment and sex, F1,101 = 6.16, P<0.05; planned comparison: male: control *vs* PE, P<0.05, **Figure 2**). To understand the efficiency of getting rewards across groups, we analyzed the proportion of (%) reinforced trials and found no group differences. However, male and female control rats reared in the enriched condition obtained more rewards than control rats reared in the standard condition. This effect was not observed in PE rats. In addition, male rats performed more reinforced trials than females (three-way ANOVA with litter as the nested factor, litter effect, 46,101 = 4.00, P<0.001, main effect of sex, F1, 101 = 18.49, P<0.001, interaction effect of prenatal treatment and rearing condition, F1,101 = 17.40, P<0.001, planned comparison: male control: standard *vs* enriched conditions, P<0.001, female control: standard *vs* enriched condition, P<0.001, **Figure 2**). The increased number of reinforced trials in control rats reared in the enriched condition was due to an overall increase in the number of total choice trials.

### Environmental enrichment altered the pattern of reaction time by increasing initiation time and decreasing movement time

**Figure 3:**
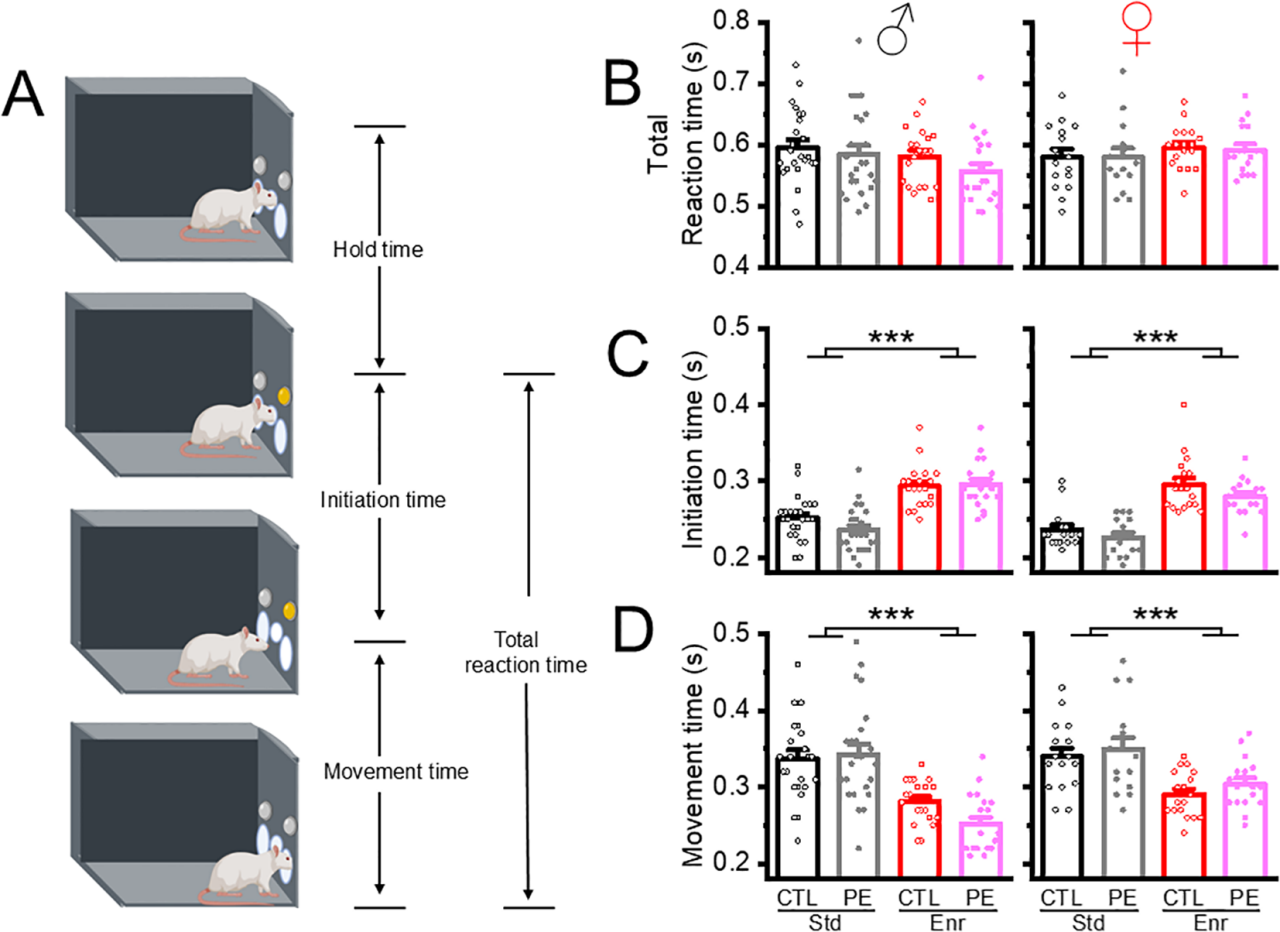
Environmental enrichment altered the reaction time (RT) pattern by increasing initiation time and decreasing movement time. The reaction time consists of two components: initiation time and movement time **(A)**. There were no group differences in total reaction time **(B)**. The initiation time was increased while the movement time was decreased in control and PE rats of both sexes reared in the enriched environment **(C, D)**. *** P< 0.001.

We analyzed three RT parameters: total RT, initiation time, and movement time to understand the speed of responding. Medians instead of means were used for these RT parameters due to their skewed distributions in each rat. The RT parameters were not influenced by hold time, which was not used as an independent variable. We did not find any group differences in total RT. Interestingly, we found the initiation time was increased in male and female control and PE rats reared in the enriched condition. In addition, initiation time was decreased in female rats compared to males (three-way ANOVA, prenatal treatment, rearing condition, sex; main effect of prenatal treatment, F1,147 = 3.93, P<0.05; main effect of rearing condition, F1,147 = 142.54, P<0.01, main effect of sex, F1,147 = 5.70, P<0.05; **Figure 3**). In contrast, movement time was decreased in both male and female control and PE rats reared in the enriched condition (three-way ANOVA with litter as the nested factor, litter effect, F46, 101 = 2.20, P<0.001, main effect of postnatal treatment, F1,101 = 67.62, P<0.001, **Figure 3**).

### Environment enrichment decreased action impulsivity in both control and PE rats

Action impulsivity was evaluated by premature initiations and false alarms. In previous studies, we observed that both parameters were exacerbated with increased cognitive load (i.e. increased hold time). The analyses were performed by dichotomizing the trials with hold times<4 s and > 4 s (5, 7). In addition, these events rarely occur when the hold time is<= 1 s. Therefore, in the present study, data analyses were performed in trials with a hold time >1 &< 4 s and > 4 s, corresponding to low and high cognitive load, respectively. To simplify the analyses and avoid high levels of factorial ANOVA, we first compared the control and PE rats reared in the standard condition, followed by analyzing the effects of environmental enrichment. We also analyzed the time for completing each trial (trial completion time) to understand how action impulsivity could impact the efficiency of completing each trial. We found premature initiations, false alarms, and trial completion time increased as hold time increased in all groups of rats. Therefore, we did not report the main effect of hold time in ANOVAs.

**Figure 4:**
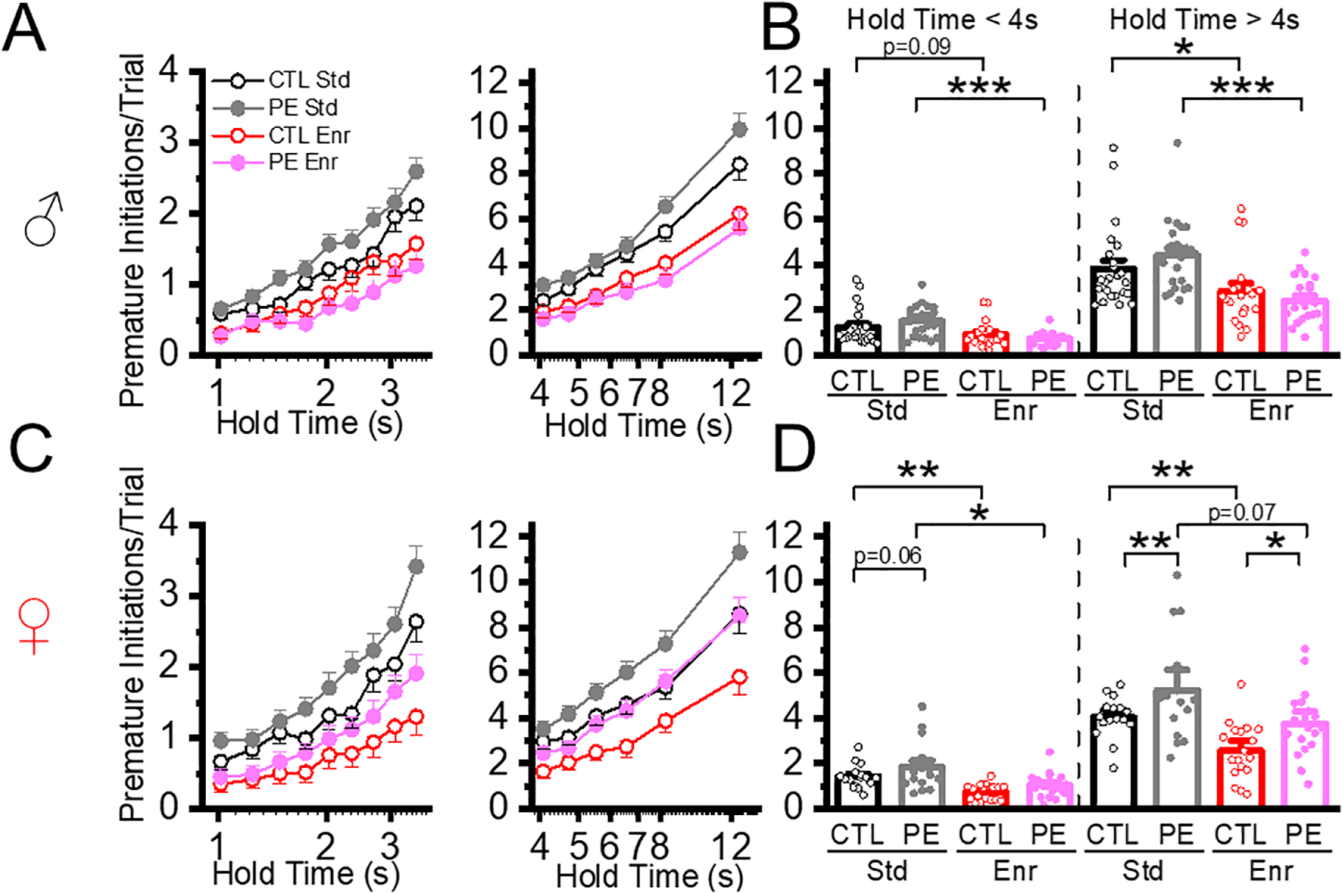
Environment enrichment decreased action impulsivity measured by premature initiations in both control and PE rats. Action impulsivity was measured by premature initiations/trial during hold time. The data was analyzed at low and high cognitive loads (hold time< 4 s and hold time > 4 s). Environmental enrichment reduced premature initiations/trials in male control and PE rats at both low and high cognitive loads **(A, B)**. Similar effects were observed in female rats **(C, D)**. Increased premature initiations/trial were also observed in female PE compared to controls but not male rats **(C, D)**. *P< 0.05, **P< 0.01, ***P< 0.001.

In trials with hold time< 4, increased premature initiation was found in PE rats in the standard condition. Sex effect was also observed. Females showed more premature initiation than males (three-way mixed-design ANOVA with litter as a nested factor, prenatal treatment, sex, hold time; litter effect, F50,101 = 1.00, P<0.01, interaction effect of prenatal treatment and hold time, F8,808 = 2.96, P<0.01, interaction effect of sex and hold time, F8,808 = 3.52, P<0.05, planned comparison: female control *vs* PE, P = 0.06. **Figure 4**). In trials with hold time > 4 s, premature initiations were increased in PE rats reared in the standard condition. Female rats also showed more premature initiation than male rats (three-way mixed-design ANOVA with litter as the nested factor: prenatal treatment, sex, hold time; litter effect F50,101 = 2.07, P<0.01; main effect of sex, F1,101 = 5.05, P< 0.05; interaction effect of prenatal treatment and hold time, F5, 505 = 6.20, P< 0.001; planned comparison: females: control *vs* PE, P<0.01, **Figure 4**).

We analyzed the effect of the enriched condition on premature initiation. We first examined the effect in trials with hold time< 4 s and found rearing in the enriched condition decreased premature initiations in both control and PE rats. Female rats had more premature initiations than males (in control rats: three-way mixed-design ANOVA, rearing condition, sex, hold time; interaction effect of postnatal treatment, sex, and hold time, F8,592 = 2.32, P<0.05; planned comparison: male control: standard *vs* enriched: P = 0.09, females control: standard *vs* enriched, P< 0.01; in PE rats: three-way mixed ANOVA with litter as a nested factor: rearing condition, sex, hold time; litter effect, F24,49 = 2.04, P<0.05, interaction effect of hold time and postnatal treatment, F8,392 = 9.92, P< 0.001, interaction effect of hold time and sex, F8,392 = 3.79, P< 0.001, planned comparison: male PE: standard *vs* enriched, P<0.001, female PE: standard *vs* enriched, P< 0.05, **Figure 4**). We next examine the enrichment effect in trials with hold time > 4 s. Rearing in the enrichment condition decreased premature initiation in control (three-way mixed ANOVA, rearing condition, sex, hold time; interaction effect of postnatal treatment and hold time, F5, 370 = 6.23, P< 0.001; plan comparison: males control: standard *vs* enriched condition, P<0.05; female control: standard *vs* enriched condition, P< 0.01, **Figure 4**) and PE rats (three-way mixed ANOVA with litter as a nested factor, rearing condition, sex, hold time, litter effect, F24,49 = 2.04, P<0.05, interaction effect of postnatal treatment, sex and hold time, F5, 245 = 5.77, P< 0.001; plan comparison: males control: standard *vs* enriched P<0.001; female control: standard *vs* enriched, P=0.07 of both sexes, **Figure 4**).

**Figure 5:**
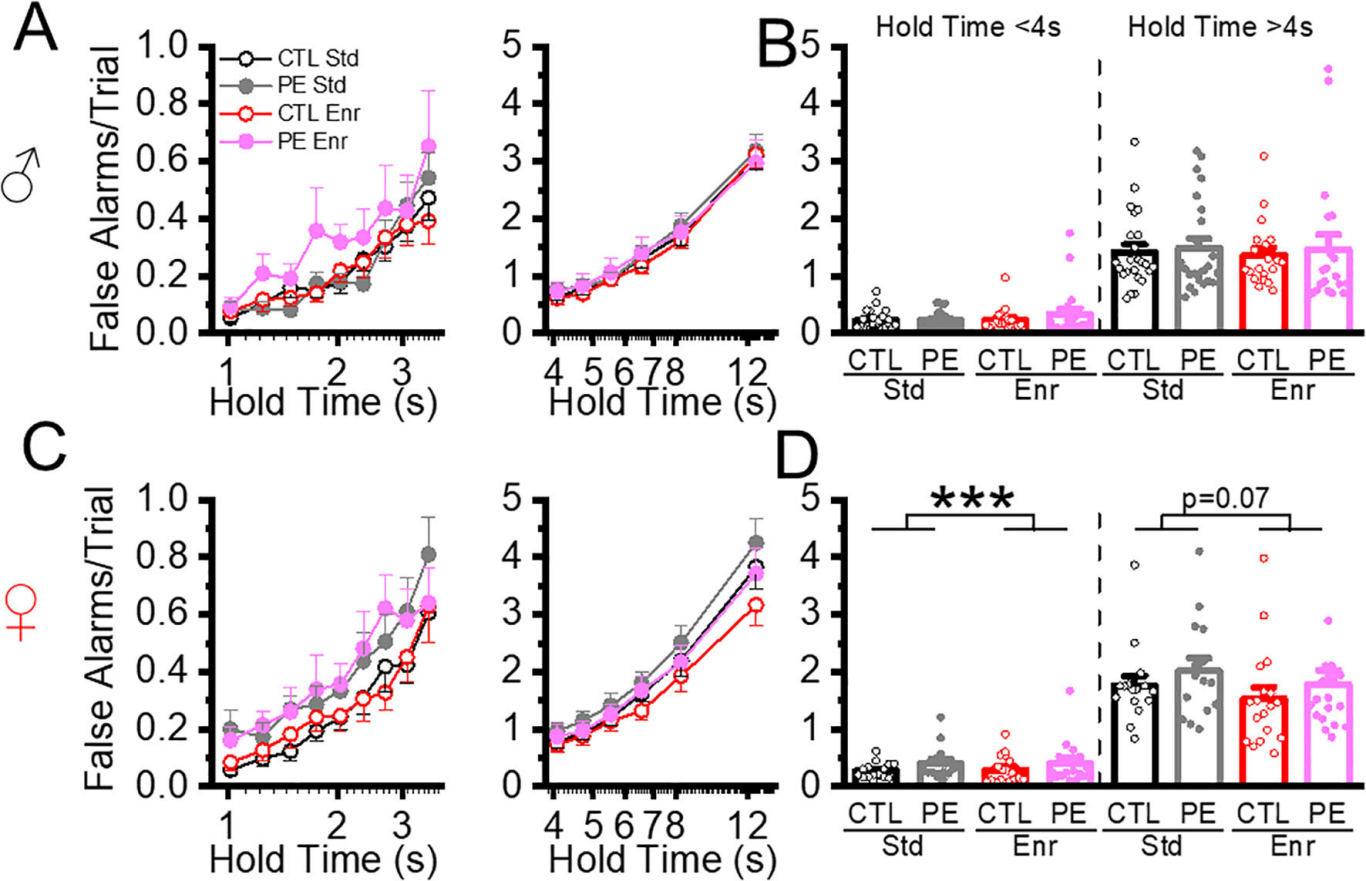
Environment enrichment did not alter action impulsivity measured by false alarms. Action impulsivity was measured by false alarms/trial at low and high cognitive load (hold time< 4 s and hold time > 4 s). We did not find a PE effect on rats reared in the standard condition **(A-D)**. Environmental enrichment reduced the false alarms/trial females with low and high cognitive load **(C, D)**. ***P< 0.001.

We next analyzed false alarms, which took place less frequently than premature initiation. In the standard rearing condition, no PE effects were found when hold time was either< 4 s or > 4 s. The only effect found was increased false alarms in female PE rats compared to male PE rats (three-way mixed ANOVA, prenatal treatment, sex, hold time, hold time< 4 s: interaction effect of sex and hold time, F5, 250 = 2.89, P< 0.05; plan comparison: PE: male *vs* female: P<0.01; hold time > 4 s: interaction effect of sex and hold time, 5,375 = 5.16, P<0.001; planned comparison: PE: male *vs* female: P<0.05, **Figure 5**). We next examined the effect of the enriched condition. In control rats, in trials with hold time< 4 s, we did not find an enrichment effect. Only sex effect was found (Three-way ANOVA, postnatal treatment, sex, hold time, interaction effect of sex and hold time, F8,592 = 2.16, P<0.05, **Figure 5**) In trials with hold time > 4 s, we found false alarms were reduced in the enriched condition in females (Three-way ANOVA, postnatal treatment, sex, hold time; interaction effect of postnatal treatment, sex, and hold time, F5,260 = 2.45, P<0.05; planned comparison: female: standard *vs* enriched, P=0.07, **Figure 5**). In PE rats, in trials with hold time< 4 s, we found enrichment increased the false alarms in females (Three-way ANOVA: postnatal condition, sex, hold time, interaction effect of postnatal conditions, sex and hold time, F8,392 = 3.01, P< 0.01, planned comparison; female standard *vs* enriched P<0.001, **Figure 5**). No group differences were found in PE rats with trial time > 4 s. Overall, no major PE or enrichment effects were found in FA.

### Environment enrichment decreased trial completion time in both control and PE rats

**Figure 6:**
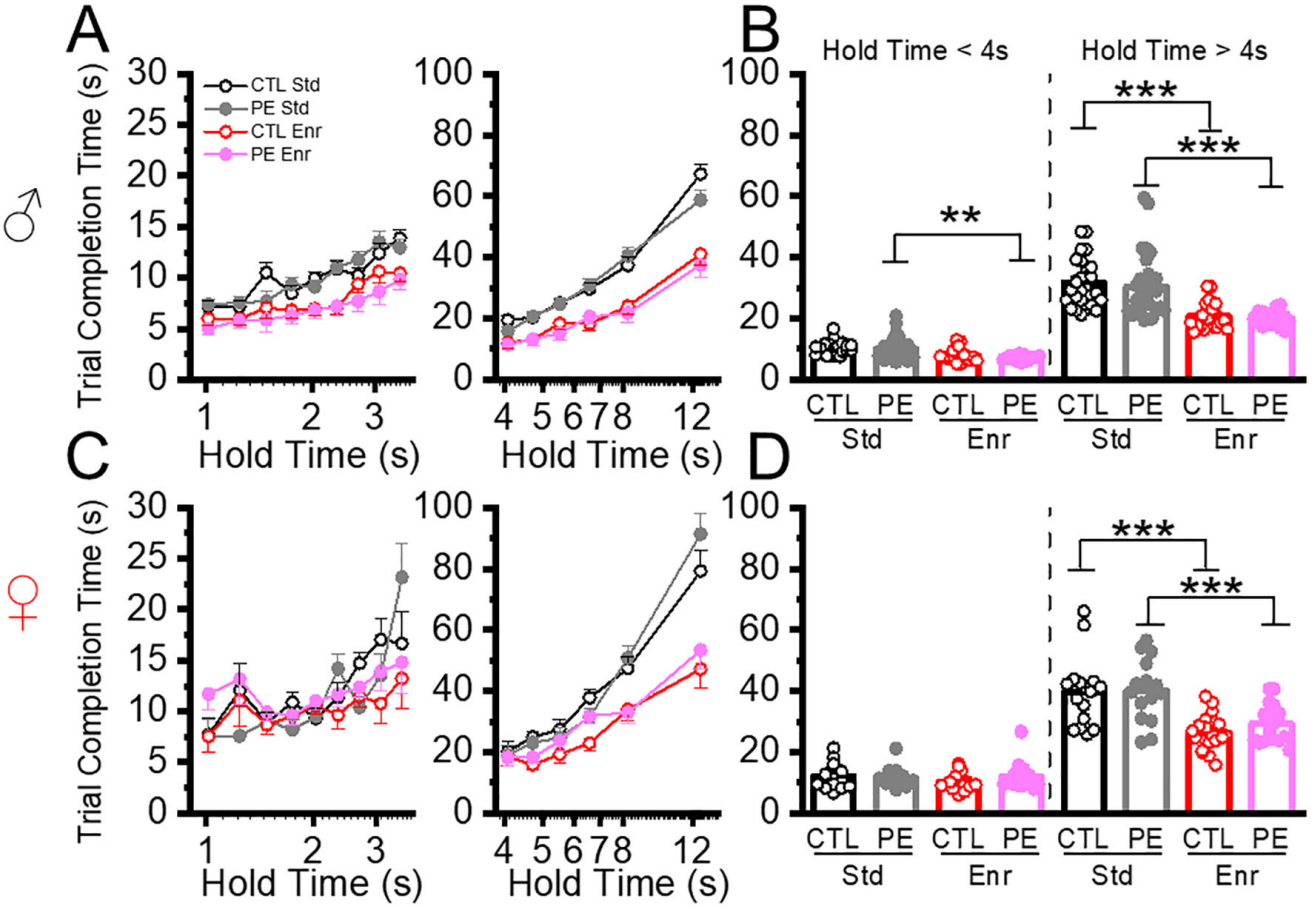
Environmental enrichment reduced trial completion time. The duration to complete each trial was analyzed at low and high cognitive load conditions (hold time< 4 s and hold time > 4 s). Environmental enrichment reduced trial completion time in male PE rats when cognitive load was low (**A, B** left panels) and females **(C, D)** control and PE rats when the cognitive load was high. **P<0.01, ***P< 0.001.

Next, we analyzed the trial completion time. In the standard condition, we did not find any PE effects for hold time< 4 s or > 4 s. We did find female rats show longer trial completion time (Three-way ANOVA, prenatal treatment, sex, hold time; hold time<4: interaction effect of prenatal treatment, sex, and hold time, F8,600 = 3.46, P< 0.001, planned comparison, PE: male *vs* female, P=0.06; hold time > 4 s: interaction effect of prenatal treatment, sex, and hold time; F5,375 = 2.75, P<0.05; planned comparison: control: male *vs* female, P=0.06, PE: male *vs* female, P< 0.05, **Figure 6**). This result revealed that longer trial completion time could be the reason for reduced trial number in females.

We next analyzed how rearing in the enriched condition could impact trial completion time. In control rats withhold time< 4 s, we did not observe an enrichment effect. Again, female rats had longer trial completion time (Three-way ANOVA with litter as the nested factor: prenatal treatment, sex, hold time; litter effect, F22,52 = 2.16, P<0.05; interaction effect of prenatal treatment, sex, and hold time, F8,416 = 2.57, P<0.01; planned comparison: control: male *vs* female, P<0.05; PE: male *vs* female: P< 0.05, **Figure 6**). In control rats withhold time > 4 s, we observed decreased trial completion time in the enriched condition in both sexes (three-way ANOVA, prenatal treatment, sex, hold time; main effect of sex, F1,74 = 8.77, P<0.01, interaction effect of rearing condition and hold time, F5,370 = 13.20, P<0.001; planned comparison: male: standard *vs* enriched, P< 0.001; female: standard *vs* enriched, P<0.001; standard: male *vs* female, P=0.07, enriched: male *vs* female, P<0.05, **Figure 6**). In PE rats withhold time< 4 s, we observed decreased trial completion time in males reared in the enriched condition (Three-way ANOVA with litter as a nested factor: prenatal treatment, sex, hold time; litter effect, F20,49 = 1.80, P<0.05; interaction effect of rearing condition, sex, and hold time, F8,392 = 2.19, P<0.05; plan comparison: male: standard *vs* enriched, P<0.01, **Figure 6**). In PE rats withhold time > 4 s, we observed decreased trial completion time in both male and female rats in the enriched condition (Three-way ANOVA with litter as a nested factor: prenatal treatment, sex, hold time; litter effect, F20,49 = 2.34, P<0.01; interaction effect of rearing condition, sex, and hold time, F5,245 = 3.22, P<0.01; plan comparison, male: standard *vs* enriched, P< 0.001, female: standard *vs* enriched, P<0.001, **Figure 6**). Taken together, the enrichment condition was effective in decreasing trial completion time in both sexes.

### Environmental enrichment decreased lapses of attention in both control and PE rats

**Figure 7:**
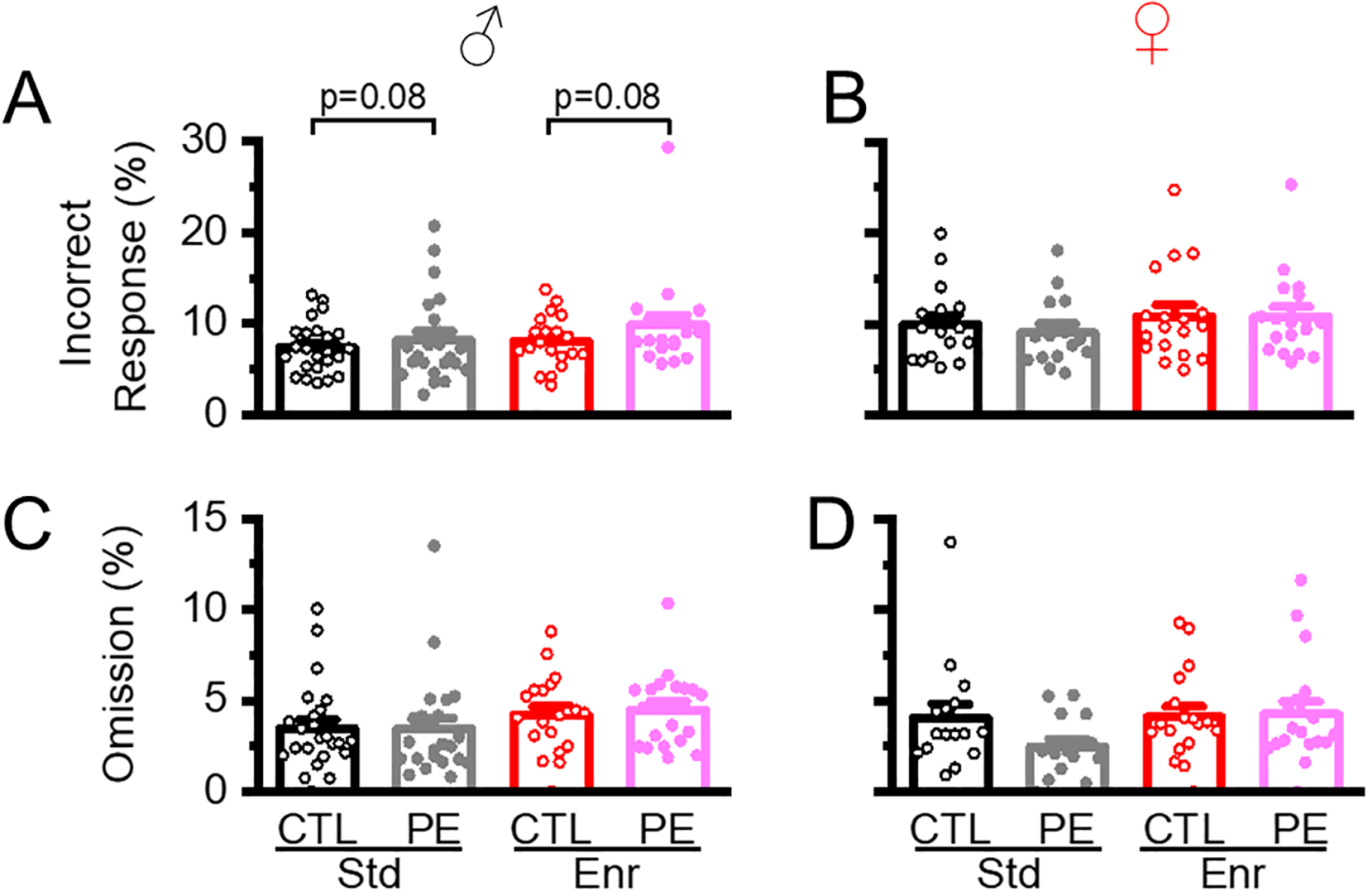
Environment enrichment did not impact lapses of attention reflected by incorrect responses or omissions. The percent incorrect response was increased in male PE rats reared in either standard or enriched condition **(A)**. No other PE or environmental enrichment effect was observed **(B-D)**.

We have used % incorrect trials and % omissions over choice trials to evaluate lapses of attention (5, 7). In male but not female rats, we found an increase in % incorrect trials in PE rats compared to controls reared in either standard or enriched conditions. No effects of environmental enrichment were found (three-way ANOVA with litter as the nested factor: prenatal treatment, rearing condition, sex; litter effect, F46,101 = 2.05, P<0.01; interaction effect of prenatal treatment and sex F1,101 = 4.81, P<0.05; planned comparison: male standard condition: control *vs* PE, P=0.08; male enriched condition: control *vs* PE, P=0.08, **Figure 7**). We next examined % omissions. No PE or rearing condition effects were found in either male or female rats (**Figure 7**).

**Figure 8:**
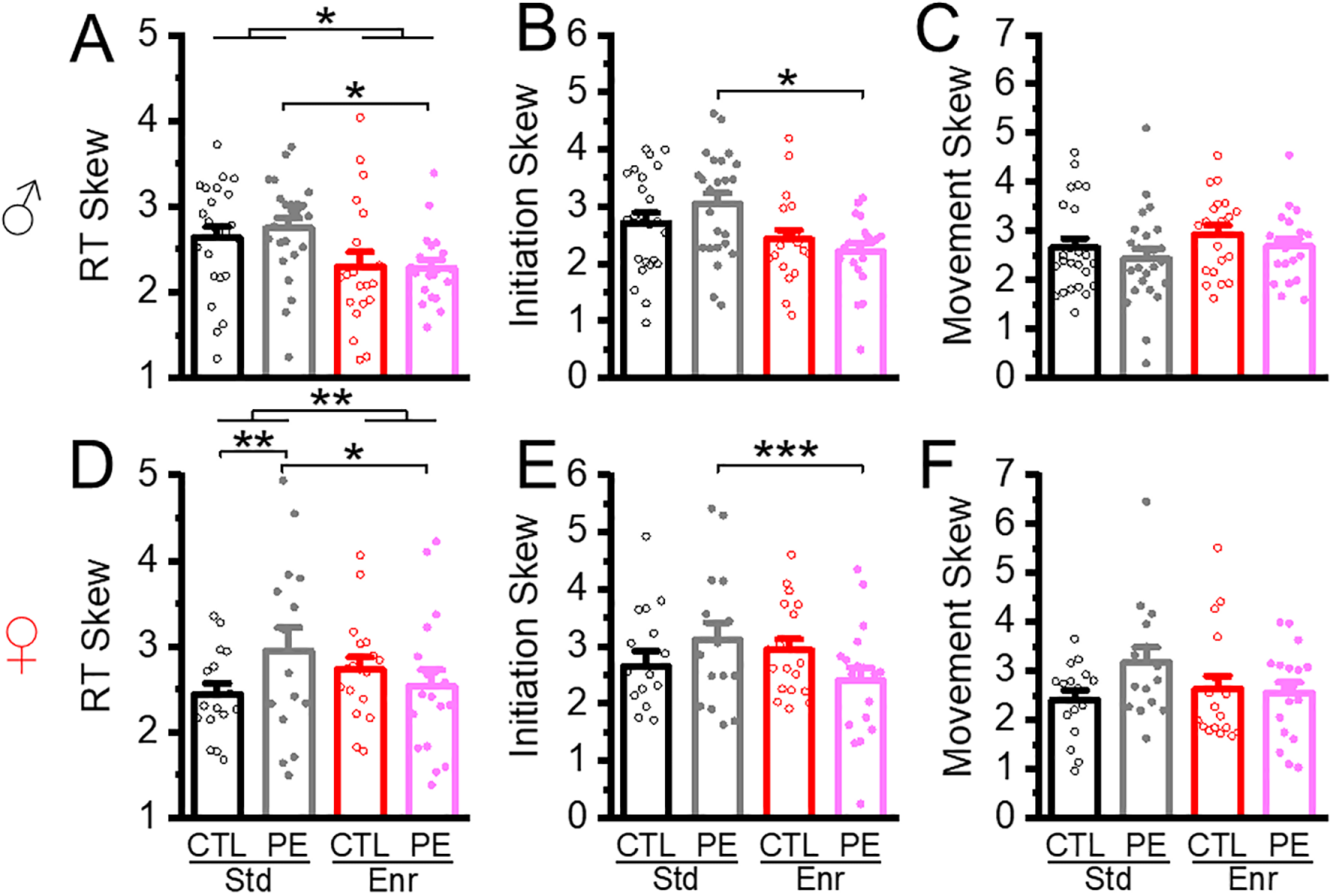
Environmental enrichment reduced the deficits in lapses of attention in PE rats. Prenatal ethanol exposure led to deficits in lapses of attention, which was reflected as increased skewness in total reaction time in female PE rats **(D)**. Environmental enrichment reduced deficits in lapses of attention reflected in total reaction time in male control and PE rats of **(A)** and female PE rats **(D)**. Environmental enrichment also decreased the skewness of initiation time in PE rats of both sexes **(B, E)**. No group differences in the skewness of movement time were found **(C, F)**. *P< 0.05, **P< 0.01, ***P< 0.001.

Lapse of attention can also be accessed by extremely large RTs, which causes positive skewness of the RT distribution (5, 7). We did not find hold times affected RTs. Therefore, all trials from each rat were used to calculate the skewness. The results showed that PE increased the skewness in females but not males in the standard condition. Enrichment decreased the skewness in both sexes (three-way ANOVA with litter as the nested factor: prenatal treatment, rearing condition, sex; litter effect, F1,46 = 1.722, P< 0.05; main effect of rearing condition F1, 101 = 5.30, P< 0.05; interaction effect of prenatal treatment and sex, F1,101 = 4.561, P< 0.05; planned comparison, female PE: standard *vs* enriched, P< 0.01, male PE: standard *vs* enriched condition: P<0.05, **Figure 8**).

We next analyzed the skewness in initiation time. Increased skewness was observed in females compared to males. In addition, the enrichment condition decreased the skewness in PE, but not control rats of both sexes (three-way ANOVA: prenatal treatment, rearing condition, sex; main effect of sex, F1,101 = 3.34, P<0.05; interaction effect of prenatal treatment and rearing condition, F1,101 = 8.18, P< 0.01; planned comparison: male PE: standard *vs* enriched condition, P <0.05, female PE: standard *vs* enriched condition: P<0.001, **Figure 8**)

Movement skewness was also analyzed. We did not find any group differences (**Figure 8**). The result shows initiation time skewness is contributing to the RT skewness.

## Discussion

The results of the present study show that rearing in an enriched environment from early development to adulthood increases the efficiency of obtaining rewards and alters the responding patterns of RT. Importantly, rearing in the enriched environment ameliorates PE-induced attention deficits, including increased action impulsivity and lapses of attention. Some of the enrichment effects are sex-dependent and observable in control rats. Taken together, rearing in an enriched environment exerts multiple beneficial effects on attentional control and task efficiency.

We have not found a PE effect on trial performance in rats reared under standard conditions. However, we find rearing in the enriched environment leads to increased choice trials in control rats of both sexes, leading to the acquisition of more reinforcers. Interestingly, this effect is not observed in PE rats reared in the enriched environment. The effect in control animals is more prominent in male than female rats, which shows a substantial escalation of trial numbers after the session starts. Increased trial numbers have also been reported in rats with environmental enrichment in a previous study (47). Such an effect cannot be attributed to more correct trials or shorter RT, which are not altered. One likely contributor to this effect is reduced action impulsivity (premature initiations), resulting in shorter trial completion time, which allows more time for the rats to perform additional trials. However, reduced premature initiations and trial completion time are observed in both control and PE rats reared in the enriched environment, which does not explain why the number of choice trials is only increased in control but not PE rats. Another likely reason is increased motivation for rewards in control enriched rats. This possibility is supported by the observation that environmental enrichment increases motivation and sensitivity toward natural rewards and influences task performance (48). These motivational factors could be confounded by PE, leading to a lack of increase in choice trials in PE rats reared in the enriched environment. Our previous studies show PE leads to disruption of the midbrain dopaminergic system support this possibility. More studies are required to examine this possibility.

Altered RT structure in rats is observed in rats reared in the enriched environment. First, neither PE nor environmental enrichment impacts the median total RT, which is the sum of initiation time and movement time. In a previous study, reduced RT has been reported in rats with environmental enrichment in a reaction time task (47). The discrepancy between Ishiwari et al. and the present study could be due to differences in data analysis and experimental paradigms. In Ishiwari et al, mean RT instead of median RT is used. Because of the skewed distribution of RT, we use median RT to better represent the RT in each animal. Second, the reaction time task in Ishiwari et al. does not involve choice. Third, the enrichment paradigm in Ishiwari et al. starts only after weaning. An interesting observation from the present study is that control and PE rats of both sexes reared in the enriched environment show slower initiation time and faster movement time. The cause of this effect is unclear. It is apparent that movement deficits are not the contributing factor because rats reared in the enriched environment show faster movement time. On the other hand, reduced action impulsivity could contribute to slower initiation time. There is a clear association between reduced initiation time and premature initiation in rats reared in the enriched condition. Furthermore, the initiation time in the present study is similar to the RT measured before motor responses in clinical studies, which report an association between RT and anxiety. Faster RT is linked to higher anxiety and less caution (49). On the other hand, environmental enrichment has also been reported to improve caution in a rat model of ADHD (50). We have found that rearing in an enriched environment significantly reduces anxiety in both control and PE rats (20). Therefore, the reduced initiation time in rats reared in the enriched environment could be due to increased caution and reduced anxiety. The faster movement time in rats reared in the enriched condition is also consistent with increased caution and preparedness. Taken together, environmental enrichment leads to a different phenotype in reaction time, probably due to increased caution and decreased anxiety.

We have observed that PE leads to increased action impulsivity indicated by augmented premature initiations, an effect that is more obvious when the cognitive load (hold time) is increased. The action impulsivity or impulsive action describes the lack of ability to control unwanted motor behavior (51, 52). Increased action impulsivity is associated with decreased inhibitory control, a key symptom of ADHD (51). We find that action impulsivity is reduced in both control and PE rats reared in the enriched environment. This effect could be a major factor leading to reduced trial time because time is not wasted on premature initiations during hold time and the interruptions within each trial. The current literature regarding environmental enrichment effects on impulsivity/inhibitory control is limited and mixed due to the following reasons. First, impulsivity is a complex construct. In preclinical studies, the two components of impulsivity, action impulsivity, and choice impulsivity involved in the decision-making process, are investigated. Environmental enrichment has been shown to reduce action impulsivity in an early study (53). On the other hand, more recent studies have shown that environmental enrichment increases action impulsivity in rodents (40) or has no effects on birds (54). The discrepancy could be caused by variability in the enrichment paradigms, age/duration of enrichment, and/or species used. In rodents, a more complex environment after weaning is associated with a reduction in action impulsivity (53, the present study), while limited environmental complexity or duration is associated with no change or increased impulsivity (40, 53) The enrichment paradigm used in the present study is comprehensive. It starts after birth (PD 2) with a short maternal separation procedure aiming at increasing maternal behavior before weaning, followed by complex housing providing social, novelty, and activity enrichment throughout the behavioral training and testing period. This approach is used because evidence shows the additive beneficial effects of neonatal handling and complex housing (39). At the present time, it is unclear how different perspectives or ages of enrichment impact action impulsivity in PE rats. Future studies are required. The information will provide critical translational information for intervention strategies for FASD.

We use the skewness of RT distribution caused by large RTs as a major index for deficits in sustained attention. We find PE leads to increased lapses of attention in female PE rats, in addition, environmental enrichment reduces the skewness of RT distribution in PE rats of both sexes but not in control rats. These results indicate that environmental enrichment can ameliorate deficits in sustained attention caused by PE. This observation is consistent with another preclinical study showing environmental enrichment could reduce inattention and improve sustained auditory attention in a rat model of ADHD using Lister Hooded rats (50).

Other than reducing attention deficits reported in the present study, evidence from preclinical studies shows multiple beneficial effects of environmental enrichment. For example, environmental enrichment can reduce emotional reactivity, enhance learning and memory, improve habituation, and increase motivation (48, 55). Using the same environmental enrichment paradigm in the present study, we demonstrated similar beneficial effects in control and PE animals. We show rearing in the enriched condition decreases anxiety and addiction risk to drugs of abuse and facilitates habituation to sensory stimuli (20, 21, 41). Among the behavioral effects of environmental enrichment, decreased anxiety could be the major moderator of attention deficits and reaction time. Previous studies, including those from our laboratory, report that PE leads to increased trait anxiety in rats (20, 24, 56–60) while reduced anxiety is observed in both control and PE rats reared in the enriched environment, resulting in no group differences (20). Indeed, previous studies have shown that environmental enrichment can effectively reduce stress and corticosterone levels (61). On the other hand, long-lasting anxiety and increased cortisol levels are associated with worse outcomes for many neurodevelopmental diseases, even in those with genetic contributions, such as ADHD (55). The inhibitory control is impacted by chronic stress (62–64). Anxiety and attention deficits, or ADHD, have a strong association in the clinical literature (25). However, the causal relationship is not clear. To understand if anxiety could exacerbate attention deficits, we have exposed rats to chronic unpredictable stress during adolescence to generate persistent trait anxiety in adulthood (26). The results show increased action impulsivity in both male and female rats, while the exacerbation of sustained attention is only observed in female rats. These observations support that anxiety could exacerbate attention deficits, and reduced anxiety in rats reared in the enriched environment may mediate, at least in part, reduced attention deficits shown in the present study. Evidence from clinical literature also supports that anxiety could impact attention deficits. Specifically, anxiety is shown to increase impulsivity, decrease the efficacy of attentional control, and impair executive function (65, 66). Specifically, Anxiety impairs inhibitory control when there are threat-related stimuli.

Although clinical studies examining environmental enrichment on ADHD symptoms are limited. Recent reviews describe that warm and sensitive parental-child interaction is negatively correlated with ADHD symptoms, and physical activity could decrease ADHD symptoms (1, 67). On the other hand, an adverse home environment is positively correlated with ADHD symptoms (1). These results show that other than genetic factors, environmental factors can clearly modulate ADHD symptoms. The results from the present study support the modulatory role of postnatal environmental factors in attention deficits by showing rearing in an enriched environment throughout development can reduce attention deficits in both control and PE rats. Clinical studies often advise that children with ADHD or FASD should be placed in an environment with limited stimuli due to their easily aroused nature and habituation deficits to environmental changes. However, it has been suggested that the externalization behaviors in ADHD are caused by under-stimulation rather than over-stimulation (48). The results from the present study support that a complex, enriched, rather than simple environment during development ameliorates ADHD-like symptoms in FASD. A complex, enriched environment also ameliorates multiple other deficits in FASD such as increased addiction risk and anxiety, as well as habituation deficits. Currently, there is a lack of effective medications for FASD (14, 16). Environmental enrichment should be considered as an important intervention strategy for FASD.

The present study focuses on the impact of positive environmental factors on ADHD-like symptoms in FASD. Other than alcohol exposure, individuals with FASD also encounter many adverse prenatal and postnatal environmental factors, such as maternal stress and undernutrition, as well as early life adversity. It is unclear to what extent these negative environmental factors contribute to deficits observed in FASD. Limited preclinical studies suggest that adverse postnatal environments can worsen emotional dysfunctions and inflammation caused by PE (57, 68). Future studies using well-designed animal models are needed to clarify the role of these negative environmental factors to cognitive/behavioral deficits in FASD. The results could provide additional insights into the intervention strategy for FASD.

## Data Availability

The raw data supporting the conclusions of this article will be made available by the authors, without undue reservation.
